# Semiconductor Gas Sensors Based on Pd/SnO_2_ Nanomaterials for Methane Detection in Air

**DOI:** 10.1186/s11671-017-2102-0

**Published:** 2017-05-04

**Authors:** George Fedorenko, Ludmila Oleksenko, Nelly Maksymovych, Galina Skolyar, Oleksandr Ripko

**Affiliations:** Taras Shevchenko National University of Kyiv, 62a Volodymyrska str., Kyiv, 01601 Ukraine

**Keywords:** Nanosized Pd/SnO_2_, Semiconductor gas sensor, Methane, Sol–gel process

## Abstract

Semiconductor sensors based on nanosized Pd-containing tin dioxide have been obtained by a sol–gel technique. Semiconductor gas-sensitive materials were characterized by transmission electron microscopy (TEM) and X-ray diffraction (XRD) methods. Influence of Pd additives on sensitivity of the sensors to methane has been studied. Temperature dependences of electrical resistance in air and sensor response to methane on palladium content for the sensors based on nanosized materials Pd/SnO_2_ have been investigated.

## Background

Natural gas is one of the main energy sources nowadays. Even the development of alternative energy could not deliver from necessity of its usage, because methane, the main component of the natural gas, is widely used in industry. Methane can combine with air explosively and is one of the greenhouse gasses. That is why, its leakages are extremely dangerous from both economy and ecology point of views. Thus, the natural gas detection is an actual problem. Different types of detectors are used for its solving [1–5]. Adsorption-semiconductor sensors are quite promising among them because of their high sensitivity, low power consumption, and small weight and sizes.

Response of the semiconductor sensor is determined by a change in chemisorbed oxygen quantity on a surface of a gas-sensitive layer in the presence of an analyzed gas. Heterogeneous catalytic oxidation of the analyzed gas takes place on the surface and leads to the chemisorbed oxygen decreasing. This change in the semiconductor surface layer causes charge carrier quantity to increase in the valence band of the semiconductor that leads to a decrease in electrical resistance [6].

The sensitivity of the adsorption-semiconductor sensors can be enhanced by increasing a rate of the analyzed gas oxidation. It can be attained by addition of catalytic active dopants. In this study, nanosized tin dioxide doped by palladium has been chosen as a gas-sensitive sensor material. Tin dioxide is known to be a chemically inert semiconductor material, and palladium is known to be one of the best methane combustion catalysts.

The aim of the study was to create adsorption-semiconductor sensors highly sensitive to CH_4_ based on Pd/SnO_2_ and investigate their properties.

## Methods

### Sensor Preparation

Nanosized tin dioxide with particle sizes 10–11 nm (obtained as described in [7, 8]) was mixed with a colloid solution of carboxymethyl cellulose to form a creamy-like paste. The paste was applied on a plate (size 2.2 mm × 2.2 mm × 0.5 mm) of the sensor (the sensor construction was presented in [9]). The next step was a drying of the sensor plates in air at 90 °C during 1 h to remove excess of water. Palladium was added to the paste by a wet impregnation technique using PdCl_2_ solutions. The concentrations of the solutions were varied from 0.21 × 10^−2^ to 35 × 10^−2^ mol/L PdCl_2_. After impregnation, the sensor plates were dried at 90 °C and then were sintered in the temperature range 20–620 °C in air to form the gas-sensitive layers of the sensors. Gas-sensitive materials and sensors based on the materials were prepared by the same procedure.

### Material Characterization

Morphology of the initial obtained nanomaterials and the gas-sensitive layer materials was studied by TEM using SELMI PEM-125K (Selmi, Ukraine) with accelerating voltage of 100 kV. For TEM study, the samples were previously dispersed in ethanol by ultrasonic treatment during 10 min. The obtained colloid solution was dropped onto a copper mesh with amorphous carbon film transparented for electron beam. Vacuum in a chamber of the TEM reached to 10^−4^ Pa. Particle size distribution analysis was performed using ImageJ software.

Phase analysis of the obtained materials was conducted using a diffractometer Bruker D8 Advance with radiation CuKα (Bruker, Germany). Particle sizes of the nanomaterials were estimated using the Scherrer equation [10]:

$$ D=\frac{k\lambda}{\beta \cdot \cos \theta}, $$

where *D* is the XRD particle size; *k* is a constant that depends on crystallite shape and is close to unity (for our calculation *k* was equal to 0.9); *λ* is the wavelength of CuKα radiation (*λ* = 1.5418 Å); and *β* is a true broadening of diffraction peak (*β* = Δ − *b*, where Δ is an experimental broadening of diffraction peak; *b* is an instrumental broadening; and *θ* is a Bragg angle.

Contents of palladium in the sensor materials were determined by X-ray fluorescence analysis using an energy dispersion X-ray spectrometer ElvaX EXS - 01 (Elvatech, Ukraine).

### Catalytic Activity Measurement

Catalytic activity of the Pd/SnO_2_ materials was studied in a flow-type reactor using methane–air mixture (937 ppm CH_4_) with a flow rate 50 ml/min. Analysis of the gas mixture components was performed by a chromatographic method using a flame ionization detector (Shimadzu GC-14, Japan). The weight of the analyzed catalyst was 200 mg. The temperature of the 10% methane conversion (*T*_10_) was taken as a measure of the catalytic activity of the samples.

### Sensor Characteristic Measurement

The sensors were aged for stabilization of their electric characteristics before measurement of the sensor response to methane. In particular, they were periodically treated by methane–air mixture (937 ppm CH_4_) for 30 s per 1 h at 405 °C during 3 days.

Temperatures of the gas-sensitive layers of the sensors were measured by an infrared radiation pyrometer Optris LaserSight (Optris, Germany).

A measure of the sensor response was taken as a ratio *R*_0_/*R*_CH4_, where *R*_0_ is a value of electric resistance of the sensor in air and *R*_CH4_ is a value of electric resistance in the mixture of air with 937 ppm of CH_4_. The gas mixture was prepared in gas balloons under pressure and certified at the Ukrainian Center of Certification and Metrology. Methane–air mixtures with lower CH_4_ concentrations (from 47 to 937 ppm) obtained through dilution generators were used to study dependence of the sensor signal on CH_4_ concentration.

To determine a value of the signal of the sensor, it was placed into a chamber and heated up in air to a definite temperature in a range of 225–405 °C. Then, the chamber was flown by air or by analyzed methane–air mixture until a constant value of the detected sensor signal had been set. The rates of flows of air and the methane–air mixture were the same and equal to 400 ml/min.

The values of the sensor signals in air or in the presence of methane–air mixture were measured in a special electric stand [11]. The sensor was sequentially connected to a load resistor. The sensor electric resistance was calculated by the formula according to the Ohm’s law for a series circuit connection:

$$ {R}_s = R\cdotp\ \left({U}_{p. s.}-{U}_r\right)\ /{U}_r, $$

where *U*_*p.s.*_ is a value of voltage supplying by power source at the sensor-sensitive layer (V); *U*_*r*_ is a value of voltage at the load resistor (V); *R* is a known value of electric resistance of the load resistor (ohm); and *R*_*s*_ is a value of the sensor electric resistance (ohm) in air (*R*_0_) and in the analyzed gas mixture (*R*_CH4_).

Two parameters of the sensors (response time (*τ*_0.9_) and recovery time (*τ*_relax_)) were used to estimate dynamic properties of the sensors. A value *τ*_0.9_ was estimated as a time required for the sensor signal to reach 90% of its equilibrium value through an injection of the analyzed gas. The recovery time (*τ*_relax_) was estimated as a time necessary for the sensor to return to 10% above the initial sensor signal in air after the analyzed gas has been released.

## Results and Discussion

**Fig. 1:**
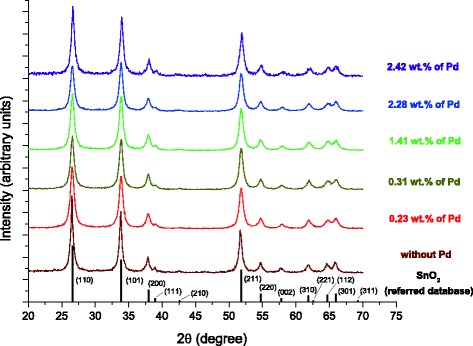
XRD pattern of sensor nanomaterials based on Pd/SnO_2_ with different palladium content in comparison with ICDD PDF-2 version 2.0602 (2006), card no. 00-041-1445 (referred database)

Previous XRD study of the obtained initial tin dioxide nanomaterial has shown a cassiterite type of SnO_2_ [8]. For the gas-sensitive Pd/SnO_2_ nanomaterials, XRD data have shown a cassiterite-type structure also (ICDD PDF-2 version 2.0602 (2006), card no. 00-041-1445) (Fig. 1); no Pd-containing phases have been found in the Pd/SnO_2_ nanomaterials because of low palladium contents in them probably.

**Fig. 2:**
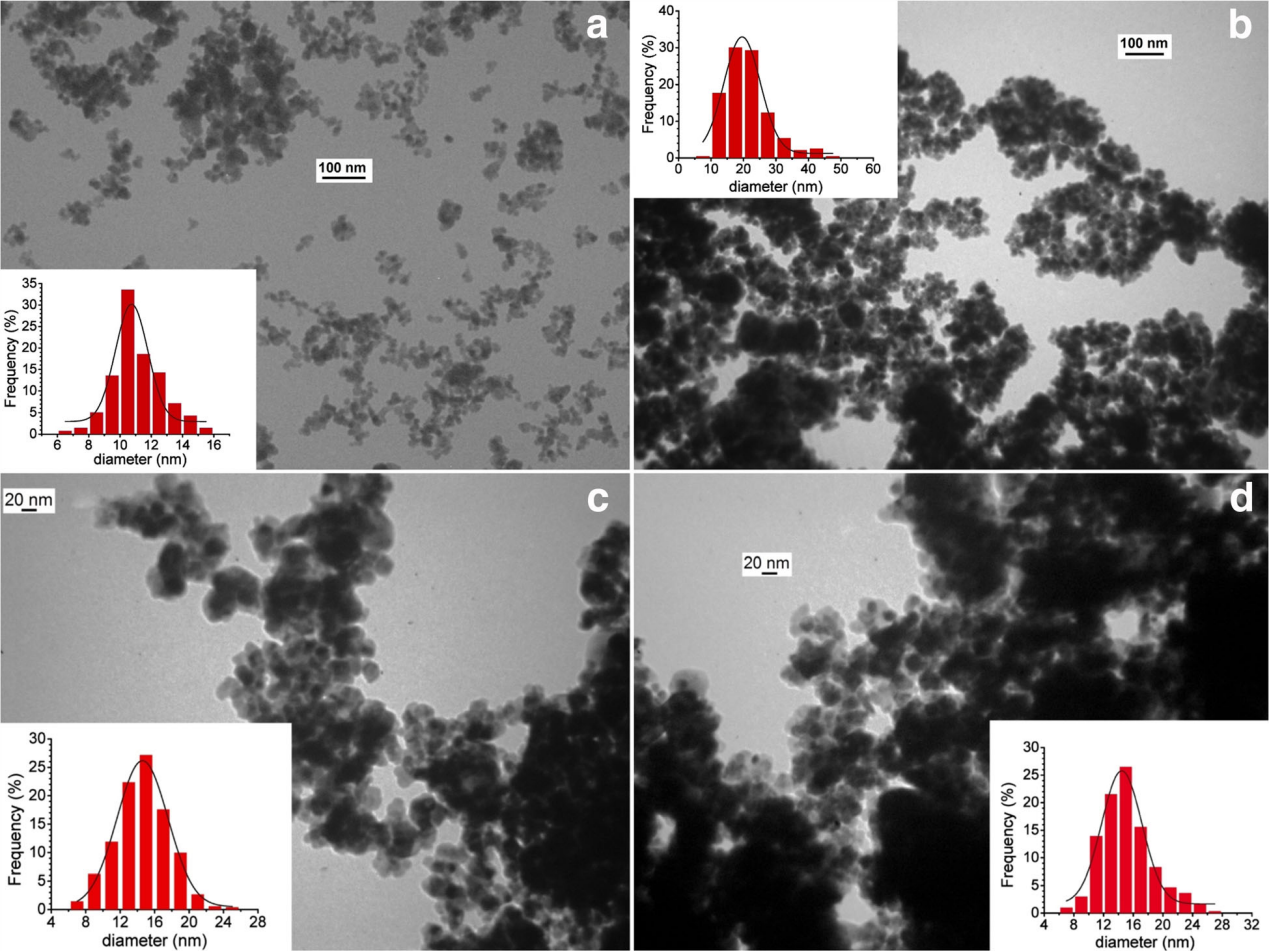
TEM images of tin dioxide and sensor nanomaterials (with particles distribution). **a** Initial tin dioxide obtained by sol–gel technique. **b** Sensor material without Pd. **c** Sensor material containing 0.23 wt.% of Pd. **d** Sensor material containing 1.41 wt.% of Pd

According to TEM images, the initial SnO_2_ sample consists of spherical particles with average sizes 10–11 nm (Fig. 2a). Thermal formation of the SnO_2_ gas-sensitive material at 620 °C in air leads to particle enlargement (average size 19–20 nm) and their following aggregation (Fig. 2b). Such enlargement can be attributed to a high final temperature of the sensor thermal treatment (620 °C). The particle enlargement and aggregation were also found for the Pd/SnO_2_ gas-sensitive materials, but their average particle sizes are 14–15 nm (Fig. 2c, d) for all investigated samples. Smaller sizes of the particles for the Pd/SnO_2_ nanomaterials in comparison with the particle sizes for the SnO_2_ nanomaterials can be explained by the stabilizing ability of the palladium additives [12].

Comparison of the particle sizes of the prepared nanomaterials determined by TEM method and calculated by XRD data (the calculation was performed by a Scherrer equation with a coefficient *k* = 0.9) has shown some differences between them (Table 1). The XRD sizes of the nanoparticles are substantially smaller than the average TEM sizes for the obtained initial tin dioxide particles. This fact can be explained by the presence of amorphous surface layers or lattice defects of the surface of the sensor nanomaterial [13, 14]. The XRD particle size is equal to the average TEM size of the particles for sensor nanomaterial on the base of SnO_2_ without palladium due to high crystallinity of the sample that is achieved by the high-temperature thermal treatment of the sensor material which leads to a significant decrease in the quantity of the structure defects. For Pd/SnO_2_ sensor materials with different palladium content, the XRD particle sizes are smaller than the average TEM particle sizes. It can be explained by two reasons. The first one is a stabilizing role of palladium during the high-temperature thermal treatment of the nanomaterials which prevents enlargement of the nanoscale particles. The second one is a formation of the additional defects in the Pd-containing nanomaterials due to the presence of palladium on the surface of the tin dioxide. In particular, the presence of defects such as dislocations and twin boundaries was confirmed for Pd/SnO_2_ systems by HRTEM method [15].

**Table 1:** Particle sizes of SnO_2_-based sensor nanomaterials obtained by TEM and calculated by XRD data

Sample	TEM size, nm	XRD size, nm
Initial tin dioxide	10–11	6.7
Sensor material without Pd	19–20	20.1
0.23% Pd/SnO_2_	14–15	12.8
0.31% Pd/SnO_2_	14–15	12.8
1.41% Pd/SnO_2_	14–15	12.4
2.28% Pd/SnO_2_	–	12.6
2.42% Pd/SnO_2_	–	12.8

**Fig. 3:**
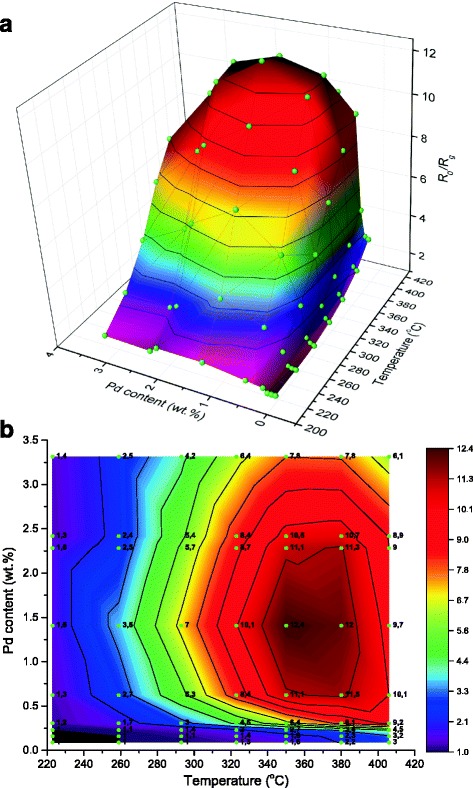
Dependences of sensor response to 937 ppm CH_4_ of the sensors based on Pd/SnO_2_ on the sensor temperature and palladium content (**a**) and their projection on “Temperature–Pd content” plot (**b**). *Balls* and *circuits* represent experimental data

To determine the optimal palladium content in the sensor nanomaterial, the responses to 937 ppm CH_4_ for the sensors based on the nanosized Pd/SnO_2_ were studied (Fig. 3). It was found that the dependences of the sensor response to 937 ppm CH_4_ on both temperature and palladium content are extremal. It can be explained by a formation of the active catalytic sites on the interface between deposited metal and support [16, 17]. In the obtained sensor materials, such interface can be formed between the palladium particles and tin dioxide support, where oxygen from air can chemisorb [18] at high temperature of the sensor [18–20] with formation of oxygen charged forms (O_2_^−^, O^−^, O^2−^).

It should be noted that electrical resistance of the sensors depends greatly on chemisorbed oxygen content. Chemisorbed oxygen species are negatively charged due to localization at chemisorbed oxygen of electrons from the semiconductor conduction band, and such process formed negatively charged surface potential. Thus, the near-surface layer of the semiconductor becomes depleted by electrons due to the presence of such a potential [21]. In the process of high-temperature formation of the sensor nanomaterial, many intergrain contacts are formed. Due to the presence of the negatively charged surface potential on each grain boundary, a lot of potential barriers are formed and they impede a directed movement of electrons in the material of the gas-sensitive layer of the sensor under an electric field action [22, 23]. As a result, a value of the electrical resistance of the sensor in air (*R*_0_) increases.

The potential barrier plays a dominant role in forming the resistance of the sensor (*R*), and when all other conditions of the sensor operation are equal (nature of the sensitive material, temperature of the sensor, etc.), a height of the potential barrier is determined by the quantity of the chemisorbed oxygen on the surface of the sensor. When the sensor is placed in air at very high temperatures, the desorption of the chemisorbed oxygen occurs [18, 19, 24], and thus, the quantity of the chemisorbed charged oxygen species decreases. This process leads to a decrease in the height of the potential barrier and an increase in the concentration of the main charge carriers in the near-surface layer of the semiconductor [21, 23]. The desorption of oxygen leads to a decrease in the sensor resistance.

In the presence of CH_4_, a chemical reaction of methane with active charged forms of oxygen occurs on the surface of the heated sensor and, as a result, the quantity of the chemisorbed charged oxygen forms decreases. Such oxygen decreasing leads also to a decrease in the height of the potential barrier in the near-surface layer of the semiconductor, and thus, the electrical resistance of the sensor decreases. A rate of CH_4_ catalytic oxidation and thus a quantity of oxygen chemisorbed on the sensor surface which takes part in the oxidation reaction depends on the methane concentration in air. The higher is the CH_4_ concentration the faster is the rate of the oxidation and the higher quantity of oxygen will take part in the oxidation reaction. As a result, a more significant decreasing in the electrical resistance of the sensor will be observed.

**Fig. 4:**
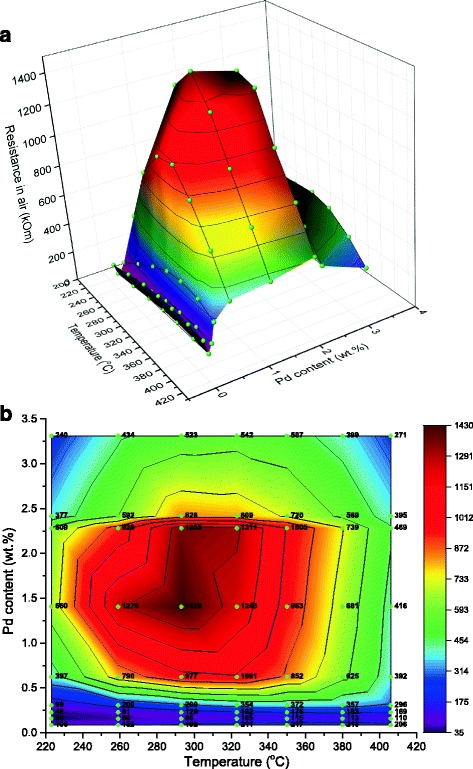
Dependences of electrical resistance in air of the sensors based on Pd/SnO_2_ on the sensor temperature and palladium content (**a**) and their projection on “Temperature–Pd content” plot (**b**). *Balls* and *circuits* represent experimental data

As can be seen from Fig. 4, the dependences of electrical resistance in air on temperature are extremal for all studied sensors. Maximums are not clearly defined (320–400 °C) for the sensors with 0.087–0.23% Pd. For the sensors based on 0.31% Pd/SnO_2_, the value of electrical resistance in air is higher and has a more defined maximum of the dependence that can be attributed to the increase in the palladium–tin dioxide length interface [24]. A further increase in the palladium content (0.62% Pd) leads to a further increase in the electrical resistance. It also makes the maximum of the temperature dependence more precise and shifts this maximum in a low-temperature region (290–320 °C). This behavior could be assigned to a change in weakly bounded oxygen quantity on the interface between the palladium particles and tin dioxide because the decrease in the palladium particle size is known to reduce the weakly bounded oxygen quantity [25]. Thus, a change in the palladium cluster sizes could provoke a change in the temperature dependences of the electrical resistances.

Concerning the temperature dependences of the sensor responses, as can be seen from Fig. 3a, b, the responses to CH_4_ for the sensors with a low content of palladium (0.087–0.31% Pd) increases with increasing temperature. The temperature dependences of the responses to methane for sensors with a high content of palladium (0.62–3.31% Pd) are extreme. Such characters of the observed temperature dependences are determined by the effect of adsorption and desorption of oxygen which takes part in the heterogeneous catalytic oxidation of methane on the surface of the sensor. It should be noted that for the sensors with palladium content more than 0.62% Pd, an increase of weakly bounded oxygen can promote a shift in the maximum of the sensor response to the region of the lower sensor temperatures as it was found for the electrical resistance of the sensors. Thus, the maximum of the responses for the sensors with 0.087–0.31% Pd could be located above 400 °C (nonexplored temperature region), but for the sensors containing 0.62% Pd, the maximum have already been observed at 350–380 °C (Fig. 3).

The highest electrical resistance was found for the sensors based on 1.41% Pd/SnO_2_ at 290 °C so the longest interface palladium–tin dioxide could be supposed for this material. Thus, the highest response to methane should be observed for the sensors with 1.41% Pd, and it was found experimentally (Fig. 3). Further increase in Pd content leads to aggregation of the palladium particles and decreases the interface length. As a result, the response and electrical resistance of the sensors based on semiconductor material with higher palladium content are lower (Figs. 3 and 4).

It should be noted that the temperature which corresponded to the maximum of the sensor response is shifted (~40–80 °C) to higher temperature in comparison with the temperature which corresponded to the maximum of the electrical resistance in air for the sensors based on the same material (Figs. 3 and 4). This shift can arise due to the factors affecting the values of the sensor electrical resistances in air and sensor response in the presence of methane at a definite sensor temperature. The value of the sensor resistance in air depends on the quantity of the chemisorbed oxygen on the sensor surface. In the presence of methane, the sensor response depends on the rate of the catalytic oxidation of CH_4_ on the gas-sensitive layer of the sensor. This rate will depend not only on the quantity of the chemisorbed oxygen but also on the temperature activation of methane that will increase significantly the oxidation rate of CH_4_ and hence the sensor response to methane. It is known that temperature activation of CH_4_ in its oxidation reaction requires high temperature for deposited systems (e.g., Pd/Al_2_O_3_, Pd/SiO_2_, Pd/SnO_2_) [12, 26, 27]. That is why, the temperature shift of the maximum of the sensor responses to methane in comparison with the maximum of their resistances becomes especially noticeable at relatively high temperatures (350–380 °C) of the sensor (Fig. 3) when the temperature activation of methane can be achieved. The effect of such activation is confirmed by the data in [12], where the high catalytic activity of deposited systems Pd/SnO_2_ in the methane oxidation reaction was achieved at high temperatures and by our data for the created Pd/SnO_2_ sensor nanomaterials also. In particular, it was found that the catalytic activities of the obtained Pd/SnO_2_ sensor nanomaterials were only observed at high catalyst temperatures (10% methane conversion is achieved at temperatures of 358–474 °C). Besides, it is known that chemical interaction of methane with charged forms of chemisorbed oxygen affects the conductivity of tin dioxide and the presence of two forms of palladium is necessary for the catalytic reaction of CH_4_ oxidation in the palladium-containing deposited systems [28]. XPS study of the obtained Pd/SnO_2_ sensor nanomaterial showed a presence of two forms of palladium with binding energies of 335.5 and 337.5 eV (Pd^0^ and Pd^2+^) [29], which caused the activities of Pd/SnO_2_ sensor materials in the methane oxidation reaction and, as a result, the high responses to methane of the sensors created on their base.

**Fig. 5:**
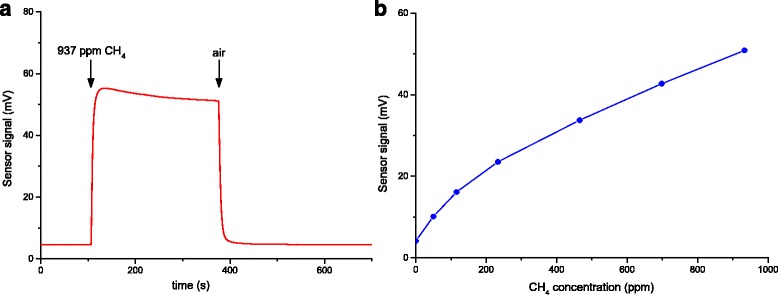
Dependence of the sensor signal on time (**a**) and on methane concentration in air (**b**) for the sensor based on 1.41 wt.% Pd/SnO_2_ at the sensor temperature 350 °C

It was found that the highest sensor response was found for the sensors with 1.41% Pd (*R*_0_/*R*_g_ = 12.4). Such response exceeds the most of those described in the literature [30–37]. Besides, the optimal sensors based on Pd/SnO_2_ are fast-acting and have a response time *t*_0.9_ = 6 s and recovery time *τ*_rel 0.1_ = 10 s (Fig. 5a). These dynamic properties are much better than for the sensors to CH_4_ known in the literature [36]. Study of the dependence of the signal value of the sensor based on Pd/SnO_2_ on CH_4_ concentration (Fig. 5b) has shown that the created sensor is capable to determine methane in air in a wide range of its concentrations (47–937 ppm).

## Conclusions

Perspectives of using the adsorption-semiconductor sensors based on nanosized Pd/SnO_2_ have been shown for methane detection in air. Addition of palladium in tin dioxide significantly increases the sensor response to methane (in ~6–7 times) in comparison with nondoped materials. The dependences of the electrical resistance in air and sensitivity to CH_4_ of the sensors with different palladium contents at different sensor temperatures were explained by an interaction between methane and oxygen chemisorbed on the interface between palladium particles and tin dioxide support. The created sensors can measure methane in a wide range of its concentration and demonstrate a fast response and recovery time.
